# Impact of physical activity and diet on colorectal cancer survivors’ quality of life: a systematic review

**DOI:** 10.1186/s12957-019-1697-2

**Published:** 2019-08-31

**Authors:** Ameera Balhareth, Mohammed Yousef Aldossary, Deborah McNamara

**Affiliations:** 1 Department of General Surgery, Colorectal Surgery Section, 2nd floor, King Fahad Specialist Hospital-Dammam, Dammam City, Saudi Arabia; 2 Department of General Surgery, Colorectal Surgery Section, Beaumont Hospital, Dublin, 9 Ireland

**Keywords:** Colon cancer, Colon neoplasm, Colorectal cancer, Colorectal carcinoma survivor, Colorectal neoplasm, Rectal cancer, Rectal neoplasm, Survivorship care plan, Survivorship plan, Survivorship program

## Abstract

**Background:**

Post-treatment management is essential for improving the health and quality of life of colorectal cancer (CRC) survivors. The number of cancer survivors is continually increasing, which is causing a corresponding growth in the need for effective post-treatment management programs. Current research on the topic indicates that such programs should include aspects such as physical activity and a proper diet, which would form the basis of lifestyle change among CRC survivors. Therefore, this study aimed to identify the impact of physical activity and diet on the quality of life of CRC survivors.

**Methods:**

We performed a systematic literature review regarding CRC survivors. We searched the Embase, PubMed, and EBSCOhost databases, considering papers published between January 2000 and May 2017 in any language, using a combination of the following subject headings: “colorectal cancer,” “colorectal carcinoma survivor,” “survivorship plan,” “survivorship care plan,” “survivorship program,” “lifestyle,” “activities,” “exercise,” “diet program,” and “nutrition.”

**Results:**

A total of 14,036 articles were identified, with 35 satisfying the eligibility criteria for the systematic review. These articles were grouped by the study questions into physical activity and diet: 24 articles were included in the physical activity group and 11 in the diet group.

**Conclusions:**

The research showed that an effective survivorship program can significantly help CRC survivors maintain good health and quality of life for long periods. However, there is a lack of consensus and conclusive evidence regarding how the guidelines for such a program should be designed, in terms of both its form and content.

## Background

Post-treatment is a critical period for cancer survivors, mainly due to the major mental and physical health implications that accompany diagnosis and treatment. Consequently, adequate post-treatment management is essential for improving the health and quality of life of survivors.

Advances in early detection and treatment, combined with the aging and growing of the worldwide population, have resulted in a marked rise in cancer survivorship [1]. Following breast, lung, and prostate cancer, colorectal cancer (CRC) is the fourth most common source of new cases of cancer and is the second-leading cause of cancer-related deaths in the USA [2]. In 2014, Siegel et al. projected that 136,830 Americans would be diagnosed with CRC that year and that 50,310 would die as a result [3].

Because of this growing population of survivors, there is an increasing need for an effective post-treatment management program. However, while there are several examples of such programs in existence, there is still a lack of universal guidelines regarding the implementation of such programs [4]. Research indicates the efficacy of programs that are implemented at the national level, which in turn indicates the applicability of setting continued support and management as a basis for quality of care for cancer survivors [5]. Further, current research on the topic indicates a necessity to include certain important elements in such programs, particularly physical activity and proper dietary measures, which would represent a significant lifestyle change for many CRC survivors.

### Objectives

Considering the above, this paper aimed to identify the impact of physical activity and diet on CRC survivors’ quality of life and to lay the groundwork for the development of guidelines for an effective survivorship program.

### Rationale

Emerging evidence has indicated that lifestyle interventions that promote healthy eating, physical activity, and weight control have significant benefits to patients’ recovery. However, research on their effect following CRC treatment, particularly on quality of life, is limited. A systematic review, such as this one, can analyze available evidence to ascertain which interventions need to be made. Further, it can help identify the appropriate tools to use to attain these benefits. A systematic review can also inform survivorship care plans and facilitate the formulation of effective post-CRC care guidelines.

## Methods

### Search strategy and data collection

This systematic review utilized various electronic databases, namely PubMed, EMBASE, and EBSCOhost. These databases facilitated the location and identification of relevant sources for analysis in the review. The electronic records reviewed were for the past decade (2000–2019), with a specific focus on the latest publications. The main search terms included were “colorectal cancer,” “colorectal carcinoma survivor,” “survivorship plan,” “survivorship care plan,” “survivorship program,” “lifestyle,” “activities,” “exercise,” “diet program,” and “nutrition.”

With the help of two assistants and a librarian, all search terms were exploded, truncated, and adjusted to align with the specific database being used. Although we did not use randomised controlled trials (RCT), our search was aligned with findings from Cochrane Highly Sensitive Search Strategy phases I–III contained in the *Cochrane Handbook for* Systematic reviews of interventions

The review did not place any language restrictions on the searches, although we limited the findings to published literature. Studies that focused on types of cancer other than CRC were excluded.

A search for the title “diet physical activity and quality of life in colorectal cancer survivors” in PubMed restricted to the last 10 years returned a total of 1502 results. The phrase “effect of diet and physical activity on quality of life of colorectal cancer survivors” was used in the EBSCOhost service, in the databases of Health Source: Nursing/Academic Edition, MasterFILE Premier, MEDLINE, Academic Search Premier, AHFS Consumer Medication Information, and Health Source: Consumer Edition. The search results contained 21,874 full-text materials. The search terms were combined a number of times, and cross-referencing was used to identify any additional articles not shown in the database search.

### Data extraction

The titles and abstracts of the selected studies were screened by the reviewers. Subsequently, the full texts of the identified studies were screened for eligibility. Two reviewers independently extracted the study attributes of eligible studies. These attributes included first author, sample size, year, country, journal, sampling, inclusion/exclusion criteria, study design, baseline response rate, comorbidities, type or timing of physical activities, type of diet, quality of life assessment approaches, statistical methods, and adjustments. Discrepancies were discussed between the reviewers, and if disagreements continued, a third independent review was asked to share his/her opinion.

### Protocol

Methods for the inclusion criteria data analysis and the inclusion criteria themselves were specified in advance and documented in a protocol [6]. See the attached protocol.

### Eligibility criteria (inclusion and exclusion)

A priori criteria were utilized for the inclusion of studies whereby we first located the abstract and subsequently proceeded to read the full-text articles if the abstracts did not offer sufficient information. In order to be included in the review, studies had to assess the quality of life of CRC survivors. Furthermore, the studies had to examine the physical activity (PA) of CRC survivors, both short term and long term, as long as specific results were provided. However, the examination of the quality of life had to exceed 2 years; otherwise, the studies were excluded. All types of colorectal cancers were eligible for the study. Since quality of life is a multidimensional concept, related studies had to conduct an assessment using more than one scale; otherwise, they were excluded. Furthermore, we included studies that investigated more than one type of cancer, although we focused on the results for colorectal cancer survivors. In all studies, PA and diet had to be independent variables and the quality of life had to be the result. All types of original quantitative studies, primarily published in English, were included. Furthermore, all studies that focused on the effect of diet and PA on the quality of life of patients with CRC, whether as individual elements or combined, were included without discrimination regarding factors such as age and language. However, the review excluded study protocols, reviews, theses, conference abstracts, qualitative studies, editorials, meta-analyses, and commentaries. The studies also had to be published between 2000 and 2019.

### Type of studies

In this systematic review, we adopted a secondary research approach for data collection and analysis. The methodological procedure comprised a systematic review of the literature on the topic: “A survivorship program for CRC survivors.” The research question was formulated, based on the topic: What is the importance of physical activity (PA) and diet for CRC survivors? The data collected from the online databases (Embase, PubMed, and EBSCOhost databases) were used to answer these questions.

### Types of participants

Only CRC survivors of any age were considered for this study; studies that specifically focused on other types of cancers were excluded.

### Assessment of risk of bias

To confirm the validity of the studies that passed the eligibility test, a pair of reviewers worked independently, and with utmost reliability, to establish and verify the authenticity of the selected articles picked to be analyzed in this systematic review.

### Assessment of risks and bias of individual studies

The two reviewers checked the quality of the methodologies for each of the studies used in the review. The items for the evaluation were adapted from a checklist formulated in Mols, et al. [6] with a particular focus on content that was essential to the specific questions in this review. Information bias was addressed through adequate assessment of study exposure, including determination of whether valid physical activity instruments were used, all aspects of physical activity, and the use of objective measures instead of self-reports. Adequate description of the data was shown, both medical and sociodemographic, such as the age of participants and their stage of cancer, including the process of data collection, whether these were self-reports or interviews.

Selection bias was addressed through the formulation of effective inclusion and exclusion criteria. Furthermore, reviewers assessed participation bias aspects, such as participant attrition, information concerning drop-outs, and non-participants, at the beginning of the review. The reviewers accommodated factors such as adequate information concerning time from diagnosis, sample size, potential study design, and correction of result measures for contradictions.

The systematic review was informed by the criteria established by the PRISMA guidelines [7].

## Results

A total of 14,036 articles were identified, with 35 satisfying the eligibility criteria for the systematic review. These articles were grouped by the study questions into physical activity and diet: 24 articles were included in the physical activity group and 11 in the diet group.

### Physical activity in CRC survivors

Of the 24 papers related to PA in CRC patients )Table 1), several reported health-related physical fitness to be effective for obtaining positive patient outcomes. Previously, PA has previously been determined to have a positive impact on the negative outcomes of cancer and its treatment, improving survivors’ quality of life [8]; however, very few existing programs can encourage cancer patients and survivors sufficiently to meet their PA guidelines [9]. Nevertheless, for CRC survivors, exercise is widely recommended as a means of improving fitness and enhancing patient-reported outcomes. PA can lead to a considerable reduction in the risk of cancer recurrence, and a previous study’s PA program was found to be successful in improving the quality of life of CRC survivors [10]. In particular, by implementing a 12-week supervised exercise training program, Sellar et al. reported that a PA approach is both feasible and effective for recurrence prevention, provided that there is a high level of participation and follow-up [11].

**Table 1:** Matrix of evidence

Study (year)	Purpose	Sample size	Study design (LOE)	Data collection method and instruments	Findings and conclusions	Comments
Anderson, Steele, and Coyle (2013)	To evaluate patients’ perceptions regarding their need for advice concerning activity and diet, as well as their belief in the role played by lifestyle in lowering the risk of cancer recurrence	40 CRC survivors	Focus group discussion	Interview schedule	The results revealed a high level of willingness among cancer survivors to obtain diet-related advice that can help them prevent symptoms	It is critical for healthcare providers to be capable of offering advice to survivors regarding suitable dietary choices
Cercek and Holt (2017)	To describe the situation of CRC survivors who do not adequately follow surveillance guidelines and who receive inadequate patient care	Not indicated	Literature review	Review of previous research	The results of the review indicated the need for gastroenterologists to improve their roles regarding coordinating the management of CRC survivors	It is necessary to include increased surveillance as part of a coordinated management program for CRC survivors
Chen et al. (2017)	To assess the sufficiency of a four-week trimodal rehabilitation program for improving elderly patients’ functional capacity following cancer surgery	116 elderly patients prepared for CRC surgery	Randomized control trial	Measurement questionnaire	The results indicated that the trimodal rehabilitation program had a positive effect on levels of PA, as well as on functional walking ability	The results indicate the potential to improve PA and physical function among elderly cancer patients
Courneya et al. (2014)	To provide an update on the Colon Health and Life-Long Exercise Change (CHALLENGE) trial, which is a three-year exercise program concerning disease-free survival	250 patients	Randomized controlled trial	Questionnaire	The CHALLENGE trial was proven to be effective as a randomized controlled trial assessing the effect of an exercise program on disease-free survival	The results indicate the need to devise similar programs to positively implement disease-free survival
Davies, Batehup, and Thomas (2011).	To update, through a review, existing literature concerning the role of diet and physical activity (PA) on cancer incidence	43 records	Literature review	Comprehensive review of the literature	The results from the review indicated that a low-fat, high-fibre diet plays a protective role in preventing the progression and recurrence of cancer	The need for a suitable diet is evident, suggesting the importance of implementing dietary interventions for cancer survivors
Devin et al. (2016)	To compare the effects of 4 weeks of moderate- and high-intensity exercise (HIE) training on the body composition of CRC survivors	47 post-treatment CRC survivors	Randomized controlled trial	Questionnaire	The results indicated that HIE, in comparison to short-term training, is a safe, feasible, and effective intervention offering clinically significant improvement in cardiorespiratory fitness as well as body composition	The findings clarify the need for effective HIE programs for CRC survivors
Doyle et al. (2006)	To summarize the findings of the American Cancer Society regarding the information-seeking behaviours of cancer survivors	Not indicated	Literature analysis	Review of research findings	The researchers indicate issues related to the availability of information concerning nutrition and PA, and the potential for patients to make informed decisions when provided with such information	The report indicates the need to provide relevant information on the nutritional- and PA-related needs of cancer survivors
D’Souza, Daudt, and Kazanjian (2016)	To investigate the sociodemographic, physically limiting, and behavioural factors that influence leisure-time PA among CRC survivors	2378 studies, conducted between 1997 and 2010	Meta-analysis	Review of previous surveys on the topic	It was revealed through multivariable regression models that compliance with PA is limited among racial minorities and individuals with ≥ 2 physically limiting enduring conditions, and also among current tobacco users	The findings indicated a need for greater efforts to increase the various patient groups’ compliance with leisure-time PA requirements
Eakin et al. (2015)	To investigate the implementation of evidence-based lifestyle interventions targeting cancer survivors	900 participants	Survey	Questionnaire	The results indicated the importance of resources and guidelines in regard to the implementation of evidence-based lifestyle interventions	Interventions for cancer survivors are critical, as a result of the various challenges they face after diagnosis
El-Shami (2015)	To review literature concerning guidelines for the management of long-term care for CRC survivors	Not indicated	Literature review	Review and analysis of research on the topic	The results indicate the importance of communication and coordination of care between oncologists and primary care physicians	It is critical that long-term care for CRC survivors is considered in post-treatment interventions
Fisher et al. (2016)	To investigate the benefits of post-diagnosis PA for CRC survivors	495 CRC patients;survey	Survey	Open-ended questionnaire	The findings indicated the benefits of PA following cancer treatment, while also identifying barriers to effective implementation	Addressing barriers to effective implementation is essential for realizing the benefits of PA
Forbes et al. (2017)	To examine how an Internet-delivered, distance-based PA-behaviour-change program affects cancer survivors’ motivation to practice PA	95 breast cancer, prostate cancer, and CRC survivors	Randomized controlled trial	Structured questionnaire	The Internet-based program was proven to have a negative impact on cancer survivors’ motivation	It is possible that the results were limited by methodological challenges, including the means of measuring levels of motivation
Golsteijn et al. (2017)	To investigate the role of PA in positively affecting the negative effects of cancer and related therapies	Not indicated	A literature study and interviews	Interview protocol	The results indicated the importance of general and cancer-specific PA as factors that improve the health and quality of life of cancer survivors	It is critical for patients to adopt a positive lifestyle that includes PA
Grimmett et al. (2011)	To examine the prevalence of health-related behaviours in CRC survivors, as well as the associations between health behaviours and quality of life.	495 CRC patients	Survey	Questionnaire	The results indicated that most cancer survivors in the UK have suboptimal health behaviours	It is critical that lifestyle change and positive health behaviours be promoted among cancer survivors
Grote et al. (2016)	To examine the feasibility of blended aerobic and resistance training (CART) as a means of improving cancer survivors’ cardiometabolic health	11 cancer survivors	Descriptive and longitudinal pilot study	Scheduled interviews	The study revealed a close relationship between CART and cancer survivors’ cardiometabolic health	The importance of implementing appropriate PA programs is underlined in this research
Ho et al. (2016)	To identify particular concerns of CRC survivors regarding various areas of their well-being, including physical, psychological, and social functioning	30 CRC survivors	Focus group discussion	Interview schedule	Participants revealed considerable dissatisfaction with the information they receive regarding their treatment	There is a need to address barriers to effective care and management of patients following treatment for CRC
Husson et al. (2015)	To examine the longitudinal relationship between HRQoL and the PA of CRC survivors	9152 cancer survivors	Telephone survey	Questionnaire	The results indicated a considerable positive relationship between PA and HRQoL. The relationship was found to be consistent up to 2 years following diagnosis	The results underlined the need for a PA program for CRC survivors
Keesing, McNamara, and Rosenwax (2015)	To focus on cancer survivors’ experiences and obtain their perspectives regarding survivorship care programs	11 qualitative studies	Literature review	Systematic review of qualitative literature	The research revealed the importance of survivorship care programs for cancer survivors	It is critical to implement effective programs that support the management of cancer survivors
Ko et al. (2010)	To establish whether the relationship between CRC intervention and fruit and vegetable consumption among survivors is mediated by information processes	266 CRC survivors	Randomized control trial	Questionnaire	Information processes, such as trust in and relevance of communications, were found to be important mediators between CRC interventions and fruit and vegetable consumption	The results indicated the importance of devising effective communication processes to increase survivors’ adherence to CRC interventions and fruit and vegetable consumption
Krouse et al. (2017)	To examine the (inadequately studied) relationships between HRQoL, PA, and bowel function in rectal cancer survivors	1063 rectal cancer survivors	Multidimensional survey	Questionnaire	Fulfilling PA guidelines was shown to have a positive relationship with HRQoL	It is essential, for CRC survivors, to develop and ensure adherence to PA in order to improve quality of life
Lawrence et al. (2017)	To identify situations in which CRC survivors participate in PA and those in which they engage in sedentary behaviours, and to use this information to influence health-promotion interventions	31 cancer survivors	Exploratory study	Structured interview schedule	The study revealed environments in which survivors of CRC are active and in which they are sedentary	The findings showed the criticality of considering the home environment when devising interventions that encourage PA and discourage sedentary behaviour
Leong et al. (2017)	To investigate the effect of follow-up and survivorship programs in regard to the management of cancer survivors	Not indicated	Literature review	Review of the literature on follow-up and survivorship programs	The results indicated the importance of effective follow-up and survivorship programs for addressing issues such as recurrence of disease	Care providers should implement effective follow-up and survivorship programs as part of post-treatment management
Ligibel (2012)	To investigate the association between diet, PA, and body weight (i.e., energy balance) and the chance of cancer recurrence and mortality in various forms of cancers	Not indicated	Literature review	Review of the literature on lifestyle behaviour	The review indicated the association between lifestyle factors and cancer prognosis	This study, regarding the management of cancer survivors, provides recommendations for further research and for necessary lifestyle changes
McGowan et al. (2017)	To discern CRC survivors’ preferences regarding PA programs and counselling	600 CRC survivors	Population-based, cross-sectional mailed survey	Questionnaire	The evidence indicated a high level of preference for participation in a PA program	The results indicate the need for PA programs that are tailored to cancer survivors
Meyerhardt et al. (2013)	To evaluate the policy and procedures developed by the American Society of Clinical Oncology	One report	Literature review	Review of findings	The society was determined to develop relevant guidelines for post-treatment management for cancer survivors	Surveillance following treatment is critical to avoid the risk of recurrence
Miller et al. (2008)	To assess the use of dietary supplements and their association with micronutrient intake and diet quality among breast cancer, prostate cancer, and CRC survivors (≥ 65 years) at five or more years post-diagnosis	753 survivors	Telephone screening interviews	Interview schedule	In terms of the use of dietary supplements, demographics, disease, and health-related factors were revealed to play a critical role in reducing nutrient deficiencies in cancer survivors, which is similar to the situation for the general population	The results indicated the potential for supplements to ensure, among cancer survivors, adequate intake of the nutrients necessary for good HRQoL
Moug et al. (2016)	To assess evidence of the viability of performance of lifestyle interventions for CRC patients, as well as the short- and long-term advantages	Meta-analysis	Systematic review of previous research	Fourteen RCTs	The reviewed randomized controlled studies revealed the benefits of lifestyle interventions for cancer survivors	The results indicated the feasibility of and need for lifestyle interventions post-cancer treatment
Pinto et al. (2012)	To investigate the efficacy of home-based PA interventions	46 patients	Randomized controlled trial	Questionnaire	PA intervention was revealed to be effective for promoting healthy lifestyles and good quality of life among cancer survivors	This evidence indicates the need for effective home-based PA interventions for the management of cancer survivors
Rock et al. (2012)	To provide a summary of findings from the American Cancer Society regarding cancer survivors’ information-seeking concerning nutrition and PA, and the role this plays in cancer survivorship	Not indicated	Research analysis	Analysis of research findings	The results indicated the importance of dietary and PA recommendations for HRQoL	It is important for healthcare providers to use recommendations to provide advice on appropriate diet and PA for cancer survivors
Sellar et al. (2017)	To examine the feasibility and effectiveness of a 12-week supervised exercise training program for CRC survivors	29 CRC survivors	Randomized controlled trial	Questionnaire	Participants engaging in the exercise training program reported better health outcomes than did the control group	The results indicate the need for an effective and feasible exercise training program to improve the quality of life of cancer survivors
Thraen-Borowski et al. (2013)	To examine, in older, long-term CRC survivors, the connection among PA, social participation, and HRQoL	1768 male and female CRC survivors	Survey	Questionnaire	The study revealed an association between PA and physical health, and between social participation and psychological health, among older, long-term CRC survivors	The results indicate the need for a comprehensive intervention program involving PA and social participation in order to improve HRQoL among CRC survivors
Vallance et al. (2015)	To determine the associations of impartially assessed, moderate-to-vigorous–intensity PA and sedentary time with psychological health in colon cancer survivors	180 colon cancer survivors	Mailed survey	Quantitative measures	The results of the study did not indicate any association between moderate-to-vigorous–intensity PA and sedentary time and depression symptoms. However, there was an association between objectively assessed moderate-to-vigorous–intensity PA and satisfaction	It is critical to establish programs that increase PA and reduce sedentary time
van Putten et al. (2016)	To examine the connection between a variety of factors (symptom- and function-related, sociodemographic, and clinical) concerning the PA of CRC survivors	9956 CRC survivors	Survey	Validated questionnaires	The study revealed a number of functioning-related and symptom-related factors that hinder PA among cancer survivors	It is essential to address the sociodemographic factors that impact the adoption of PA among CRC survivors
Winkels et al. (2016)	To assess CRC survivors’ level of compliance with eight recommendations of The World Cancer Research Foundation/American Institute for Cancer Research regarding PA, diet, and body weight	1774 CRC survivors	Survey	Questionnaires	The results indicated a low rate of adherence to the recommendations; only 12% complied with six or more of the eight recommendations, 65% adhered to between five and six; and 23% adhered to four or less	There is room for improvement in lifestyle choices in order to ensure greater adherence with recommendations concerning diet, physical exercise, and body weight

Some studies have examined how physical exercise can generate positive outcomes. Devin et al. reported that, for CRC survivors, high-intensity exercise comprises a safe, effective, and feasible intervention, because of the role it plays in improving both cardiorespiratory fitness and body composition [12]. A study by Vallance et al. showed that moderate-to-vigorous–intensity PA is critical for improving quality of life following treatment for colon cancer and that PA programs are significant in improving psychological health [13]. Moreover, Husson et al. provided further evidence that improved PA provided a positive contribution to health-related quality of life (HRQoL) [14].

Among CRC survivors, the environments within which PA interventions are implemented play a role in their efficacy and their ability to reduce detrimental sedentary behaviour [9]. One study found that, for such individuals, home-based PA interventions have a high level of efficacy in creating motivation for maintaining health-related physical fitness [15]. In accordance with the evident benefits of physical exercise, in terms of fighting and preventing cancer and its recurrence, computer-tailored interventions have been explored in relation to their potential to induce increased adherence to physical exercise; consequently, such programs have been found to show a potential to increase motivation among prostate and CRC patients and survivors [9].

A study was conducted to establish the impact of an Internet-delivered, distance-based PA program on the health and fitness of patients. It was consequently determined that motivation toward behaviour change declined when patients relied on the Internet for their physical exercise instruction. However, live PA programs delivered via the Internet are a common mode of providing self-management after successful cancer treatment. Forbes et al. studied the levels of motivation associated with such programs; they not only established that such programs have negative effects on motivation, but also that, if supervised, some programs become more effective and the rate of follow-up and participation increases [16].

However, although research findings remain inconclusive, there is evidence that information dissemination can have a positive impact on programs [17]. Brown et al. criticized the existing level of information provision concerning the onset of CRC and means of effective management following treatment [18]. Further, as mentioned, a home-care-based program has the potential for reduced efficacy as a result of reduced motivation. Considering this, Foley et al. proposed the use of a community-based multimodal exercise program [19]. Such a program can have positive impacts through its potential to improve motivation and participation. For instance, among elderly cancer survivors, along with the association between PA and improved physical health outcomes, it has been determined that such individuals’ mental health needs can be addressed through social participation. Social participation has also been revealed to promote engagement in PA [20].

One study indicated the efficacy of a combination of physical exercise and counselling in producing better outcomes in CRC survivors [21]. The nature of training programs used with cancer survivors has remained a focus of research in an effort to devise the most effective strategy for fulfilling the needs of survivors. For instance, Grote et al. investigated the efficacy of combined aerobic and resistance training for cancer survivors [22]. They found that improvement in cardiometabolic health is critical for preventing comorbidity among cancer survivors; in fact, it is as important as monitoring for the possible recurrence of cancer [22]. Further, Ligibel indicated the need to control for “energy balance factors, including diet, PA, and body weight,” in order to reduce the risk of cancer recurrence following treatment [23]. The above findings indicate the need for an effective exercise program, which utilizes PA in an appropriate manner to achieve positive results in the quality of life of cancer survivors.

In an evaluation of the Colon Health and Life-Long Exercise Change (CHALLENGE) trial, Courneya et al. confirmed the positive impact of PA on colon cancer patients [24]. Meanwhile, another study found that, in terms of CRC survivors, HRQoL is a major outcome of adherence to PA guidelines [25]. There is a lack of research on survivorship care programs for CRC survivors; hence, a more comprehensive examination of the implementation and scope of such programs, which would improve the management of care for cancer survivors, has been advocated [26]. Further, despite the limited evidence concerning the efficacy of leisure-time PA, it has been clearly determined that such interventions should be promoted among racial minorities, individuals with more than two physically limiting chronic ailments, and individuals who are currently smoking [27]. Because PA is a critical part of the post-treatment management program, factors that hinder effective PA should be identified and addressed [28]. A notable obstacle revealed in previous research is patients’ sociodemographic factors; consequently, future programs should take into consideration functioning elements, cancer symptoms, and the nature of treatment [29]. Further, inadequate information relating to treatment and management, distress from toxicities, and challenges adjusting to life and colostomy care, are other issues that should be addressed to achieve effective management of CRC survivors [30].

### Nutrition in CRC survivors

Eleven papers met the inclusion criteria for diet and CRC (Table 1). Several of these studies revealed a close relationship between a poor diet and the development of different kinds of cancer, including CRC. It is well established that an unhealthy lifestyle—poor dietary and physical fitness habits—contributes to the development of cancer [31]. Based on this, Bazzan et al. highlighted the importance of investigating the ideal diet for cancer survivors in order to improve their health and quality of life [32]. Dietary factors involved in the development of cancer can be modified to achieve positive outcomes in both treatment and post-treatment management [33]. For instance, Grimmett et al. provided a summary of the effect of modifiable lifestyle factors pre- and post-diagnosis [31]. They determined that an appropriate diet, in conjunction with PA, is critical for the management of cancer throughout the continuum of the disease. This finding is indicative of the interplay between modifiable lifestyle factors and the prognosis of the disease. Their study indicates the importance that patients adopt the recommended lifestyle, which would lead to effective management and better prognosis among CRC survivors [31].

In addition to an exercise program, nutrition has been shown to have a huge impact on the health and well-being of CRC survivors. A change in lifestyle, which includes adherence to a recommended healthy diet, has been determined to have the potential for short-term benefits among CRC survivors, indicating the need for more research and a standard program to reveal the potential long-term effects [34]. Consequently, Grimmett et al. explored the role that such a change in lifestyle plays in terms of the long-term health and survival rates of cancer survivors [31], finding that a change in lifestyle is associated with longer life and better quality of life among the CRC patient population.

Among the health-related behaviours explored in Grimmett et al.’s study is consuming a diet recommended by a healthcare provider [31]. Grimmett et al. concluded that the failure to adopt healthy behaviours is a major contributor of poorer health and quality of life among cancer survivors [31]. Further, Ko et al. replicated the findings of Brown et al. by investigating the efficacy of communication strategies in improving adherence to fruit and vegetable consumption among CRC survivors [34]. Ko et al. consequently found that, similar to the fact that the involvement of a healthcare provider increases adherence to PA programs, the enhancement of communication and trust between care providers and patients mediates adherence to nutritional programs [34].

Previous evidence has indicated that, in addition to PA, a healthy diet plays an important role in reducing the risk of developing CRC [33]; thus, such an approach can be considered to contribute to reducing the risk of recurrence following successful treatment. However, despite the relatively large amount of evidence concerning the effect of diet on primary prevention, research regarding the role of diet in secondary prevention remains inadequate. Nevertheless, it can be determined from the above findings that survivors’ lifestyle behaviours are critical for successful secondary prevention and good quality of life following treatment [31]. Several of the studies examined showed that nutrition plays an important role in terms of enhancing quality of life; furthermore, a positive relationship was identified between survival/prevention of recurrence of CRC and consuming nutritious foods, PA, and physical fitness. Additionally, a study by Miller et al. [35] reported that intake of supplements among cancer survivors is a critical intervention for satisfying the body’s nutritional demands; the researchers also underlined the importance of communication between care providers and patients.

Studies have also investigated particular diets that CRC survivors should avoid in order to achieve positive outcomes in terms of health and quality of life [30]. Consequently, recommendations regarding foods that should be avoided, including red meat and low-fibre foods, have been proliferated. Such research adds to the findings of Grimmett et al., who suggested that high-fibre fruits and vegetables contribute to preventing the recurrence of CRC [31]. Thus, lifestyle modification aimed towards promoting health and good quality of life should include avoiding foods that can place a CRC survivor at risk of recurrence. According to Ho et al., the main type of diet to avoid is the Western-pattern diet, which has traditionally been associated with causing different types of cancers, including CRC [30]. Borresen et al. sought to identify specific dietary interventions suitable for inclusion in a CRC survivorship program [36]. In particular, they examined the role of dietary fibre in the prevention of cancers of the digestive system; navy beans and rice bran were the focus of their investigation, as these have previously been suggested to restrain colon carcinogenesis. Borresen et al. consequently determined that intake of whole grains and legumes is critical for survivors of CRC because this increases levels of dietary fibre, and contributes to CRC chemoprevention [36]. However, Hawkins et al. also indicated the importance for patients to possess awareness and a positive belief stance in regard to observing a healthy diet [37]. Nutritional interventions are highly recommended to include as elements of survivorship programs for CRC survivors, because of their potential to improve the general health of such individuals [38]. Nutritional interventions, as part of behaviour-modification programs, have been found to augment the health and quality of life of survivors [30]. Applying the Theory of Planned Behaviour and the Health Action Process Approach, Moug et al. indicated the positive role of adopting positive health behaviours, including consuming a healthy diet and avoiding behaviours that put the individual at risk of cancer recurrence [39]; they also recommended CRC survivors to implement dietary interventions that are based on recognizing the role that poor dietary behaviours play in CRC [39]. Additionally, Lawn et al. recommended the inclusion of nutritional interventions in self-management strategies for improving CRC survivors’ health and quality of life [38].

Winkels et al. [40] supported the findings of Hawkins et al. [37], suggesting the important role of patients’ awareness and positive belief regarding the implementing of positive dietary behaviours. They stated that patients are most likely to adhere to nutritional interventions when they understand the importance of these interventions. Winkels et al. also investigated a sample of CRC survivors’ compliance with The World Cancer Research Foundation/American Institute for Cancer Research’s (WCRF/AICR) for CRC survivors [40]. They reported that patients with knowledge of the recommendations had higher adherence rates to their diets. Considering the above, we find substantial evidence that advice on proper dietary behaviour should form a part of a survivorship program for CRC survivors. Informed individuals are more likely to have better adherence scores and, therefore, have better outcomes in terms of health and quality of life.

The role of PA and diet was tested by applying the four risk of bias items criterion; the results showed that there is an effective survivorship program that can help CRC survivors maintain good health and quality of life for long periods. A positive association emerged from outcome assessors, concealment allocation, sequence generation, and blinding of patients.

## Discussion

For many CRC survivors, adherence to PA and dietary recommendations necessitates changes in their behaviours and lifestyles. Positive behaviour changes, such as adopting an appropriate diet and engaging in PA, have been shown to have a major impact on the outcomes of cancer survivors. However, it is important to note that, for successful behaviour change, the structure of a lifestyle-change program is critical. Studies have shown that a dogmatic approach may cause self-blame and stigmatization, while a more customized and evidence-based approach can have a positive impact [41].

Part of the mission of the American Cancer Society is to focus on informed decision-making for cancer survivors and their families. Critical information concerning diet and PA is important for positive outcomes [38]. A high level of adherence to the WCRF/AICR’s eight recommendations—regarding diet, PA, and body weight—has been shown to correlate with a tendency to change lifestyle and adapt positive dietary behaviours [42].

Self-care is critical for cancer survivors, but there is a need for better integration of lifestyle change into standard models of post-cancer-treatment care [43]. The benefits of conducting follow-ups and advocating lifestyle changes, as part of survivorship programs for CRC patients, have been widely highlighted in research (such benefits include identification of the potential for cancer recurrence and patients’ adoption of preventive measures) [44]. Thus, coordinated management of CRC survivors is essential for improving patient care, preventing recurrence of the disease, and improving general quality of life [45]. Coordination and communication between care providers and patients, in terms of addressing individual needs, are the most critical factors for effective post-treatment management of CRC survivors [46]. Consequently, an evidence-based coordinated program for cancer survivors, based on nutritional and PA needs, is essential. Additionally, healthcare providers should be provided with the necessary information to perform effective management of survivors [47].

### Limitations of the study

This systematic review gives an integrated account of several studies, which can be used to determine an effective survivorship program to help CRC survivors maintain good health and quality of life for long periods. The most prominent limitation of this systematic review is that varieties of diet, patient populations, outcome definitions, and type and frequency of physical exercises are not the same across studies. Publication bias might also have influenced some of the results.

## Conclusions

The findings of this systematic review have shown that there is a connection between effective survivorship programs for CRC survivors, and how such programs confer long-lasting benefits to this group. Conversely, the results might have been affected by reviewing imprecise studies that suggest the possibility of confounding factors other than diet and physical exercises as influencing CRC survivorship. Further research to establish if there are other factors that affect survivorship in this group is therefore warranted.

## Data Availability

All data analyzed during this study are included in this published article.

## Abbreviations

CRC: Colorectal cancer
HRQoL: Health-related quality of life
PA: Physical activity
